# Assessing Standardisation in Platelet-Rich Plasma (PRP) Injections for the Management of Greater Trochanteric Pain Syndrome (GTPS): A Systematic Review of the Available Literature

**DOI:** 10.7759/cureus.74690

**Published:** 2024-11-28

**Authors:** Uday Mahajan, Andreas Papaleontiou, Mohamed A Imam, Ansar Mahmood

**Affiliations:** 1 Trauma and Orthopaedics, Queen Elizabeth Hospital Birmingham, Birmingham, GBR; 2 Trauma and Orthopaedics, Trauma Surgery, Ashford and St Peter's Hospitals NHS Foundation Trust, Ashford, GBR

**Keywords:** gluteal tendinopathy, gtps, hip pain management, musculoskeletal disorders, platelet-rich plasma, prp injections, prp preparation methods, prp standardisation, tendinopathy treatment

## Abstract

Greater trochanteric pain syndrome (GTPS) is a prevalent musculoskeletal condition characterised by lateral hip pain and reduced function. Platelet-rich plasma (PRP) injections have gained attention as a potential treatment due to their regenerative properties. However, variability in PRP preparation methods and insufficient standardisation in the literature complicate the evaluation of its efficacy and reproducibility. This systematic review aims to assess the level of standardisation in PRP injection protocols for GTPS, focusing on preparation methods, injection techniques, and reported outcomes. A systematic review was conducted using comprehensive searches of major databases. Inclusion criteria targeted randomised controlled trials (RCTs) evaluating PRP for GTPS in adults. Four eligible RCTs were identified, and data were extracted on PRP preparation methods, injection protocols, and reported outcomes. The risk of bias was assessed using the Cochrane Risk of Bias 2 (RoB 2) tool. The included studies demonstrated significant heterogeneity in PRP preparation methods, including centrifugation speeds (1,100 gravitational force (g) to 3,850 revolutions per minute (rpm)), blood volumes (25-54 mL), and platelet concentrations (9.23 × 10⁹/L to 1232 × 10⁹/L). Injection sites varied from the gluteal tendons to the trochanteric bursa, with volumes ranging from 4 mL to 7 mL. Only one study conducted ultrasound-guided injections into the tendon. Despite the variability, two studies reported significant improvements in pain and function, while two found no difference compared to the control. This review highlights the lack of standardisation in PRP preparation and injection protocols for GTPS. Standardised guidelines are urgently needed to improve comparability across studies and optimise clinical outcomes. Future research should establish consensus on PRP preparation, classification, and reporting standards to advance its clinical application.

## Introduction and background

Greater trochanteric pain syndrome (GTPS) is characterised by persistent lateral hip pain and is frequently misdiagnosed as other conditions, such as trochanteric bursitis. Diagnosis is often challenging, as GTPS symptoms overlap with other sources of hip pain. Key features include tendinopathies with or without tears, inflammation of the bursa, and effusion, which can severely impact patients' quality of life, often to a greater extent than hip osteoarthritis [1-3].

The aetiology of GTPS involves repetitive use, trauma, and, less commonly, infection or crystal deposition. If left untreated, GTPS can progress from tendinopathy to partial or full-thickness tears, often accompanied by muscle atrophy visible on MRI [4,5]. Psychosocial factors and pain modulation also contribute to its complexity, necessitating a multidisciplinary approach to treatment [6-9]. GTPS affects 10-25% of the general population, with a higher prevalence among women aged 40-60, and it frequently coexists with conditions such as low back pain, osteoarthritis, and obesity [10,11].

Historically, GTPS was managed with conservative treatments, such as rest, ice, anti-inflammatory medications, and corticosteroid injections [12]. Contemporary approaches now include graded exercise programs, shockwave therapy, and injections, with surgical intervention reserved for persistent cases [13-16]. While acute tendinopathy often responds well to conservative management, advanced cases, such as those with full-thickness tendon tears, frequently require surgery due to the ineffectiveness of nonoperative treatments [17-19].

Platelet-rich plasma (PRP) injections have emerged as a promising treatment for higher-grade and refractory tendinopathy. PRP is an autologous preparation with regenerative and anti-inflammatory properties, facilitating tissue healing through growth factors such as epidermal growth factor (EGF), platelet-derived growth factor (PDGF), transforming growth factor (TGF), insulin-like growth factor (IGF), vascular endothelial growth factor (VEGF), and basic fibroblast growth factor (bFGF) [20-24]. Despite mixed efficacy in treating musculoskeletal conditions such as plantar fasciitis, knee osteoarthritis, and patellar tendinitis, PRP shows potential for addressing GTPS. However, conclusive evidence remains limited, and challenges such as variability in PRP preparation methods hinder its clinical application [24-30].

Given the significant variability in PRP preparation and the lack of comprehensive reporting in existing studies, there is an urgent need to assess the level of standardisation in PRP use for GTPS. Laboratory studies have highlighted inconsistencies in platelet and cell concentrations resulting from differences in PRP kits, preparation methods, and centrifugation protocols, all of which directly impact treatment efficacy [31]. Furthermore, insufficient reporting of essential parameters such as platelet concentrations and activation methods complicates the comparison of study outcomes [32]. Patient-specific variability in platelet and growth factor concentrations adds another layer of complexity [33], emphasising the need for a systematic review to critically evaluate the current state of PRP protocol standardisation in GTPS management.

## Review

A systematic review was conducted in December 2023, following the Preferred Reporting Items for Systematic Reviews and Meta-Analyses (PRISMA) guidelines. Two independent reviewers performed a comprehensive literature search across multiple databases, including PubMed, EMBASE, MEDLINE Ultimate, Complementary Index, CINAHL Complete, Academic Search Index, Gale OneFile: Health and Medicine, ClinicalTrials.gov, Directory of Open Access Journals, and Supplemental Index. The search strategy used a combination of keywords to capture studies related to GTPS and PRP injections, focusing on terms such as "greater trochanter pain syndrome", "gluteal tendinopathy", "platelet-rich plasma", and "randomised controlled trial". No date restrictions were applied, and studies in English were included to minimise language bias. Duplicate studies were removed using a reference management tool.

The screening process involved an initial review of titles and abstracts, followed by a detailed assessment of full-text articles. Inclusion criteria required studies to be randomised controlled trials (RCTs) evaluating PRP injections for GTPS in adults, with follow-up data on at least one outcome measure related to pain or function. Non-randomised studies, reviews, and studies with overlapping or incomplete data were excluded. Disagreements between reviewers were resolved through discussion or arbitration by a third reviewer. Bibliographies of included articles were screened to identify additional relevant studies, ensuring comprehensive coverage of eligible literature. The study selection process adhered to PRISMA guidelines and is visually represented in a flow diagram.

To assess methodological quality, the included studies were evaluated using the revised Cochrane Risk of Bias 2 (RoB 2) tool for randomised trials, which examined five domains: bias from the randomisation process, deviations from intended interventions, missing outcome data, outcome measurement, and selection of reported results [34]. Each study was categorised as having low, some concerns, or high risk of bias. Data extraction was performed systematically using a structured template. Extracted data included details on PRP preparation, such as blood volume, anticoagulant type, centrifugation parameters, platelet activation methods, and final PRP composition, as well as injection protocols, delivery methods, and follow-up outcomes. Outcome measures included pain scores, functional assessments, and adverse events, which were compiled into a comprehensive data table for comparative analysis across studies.

**Figure 1:**
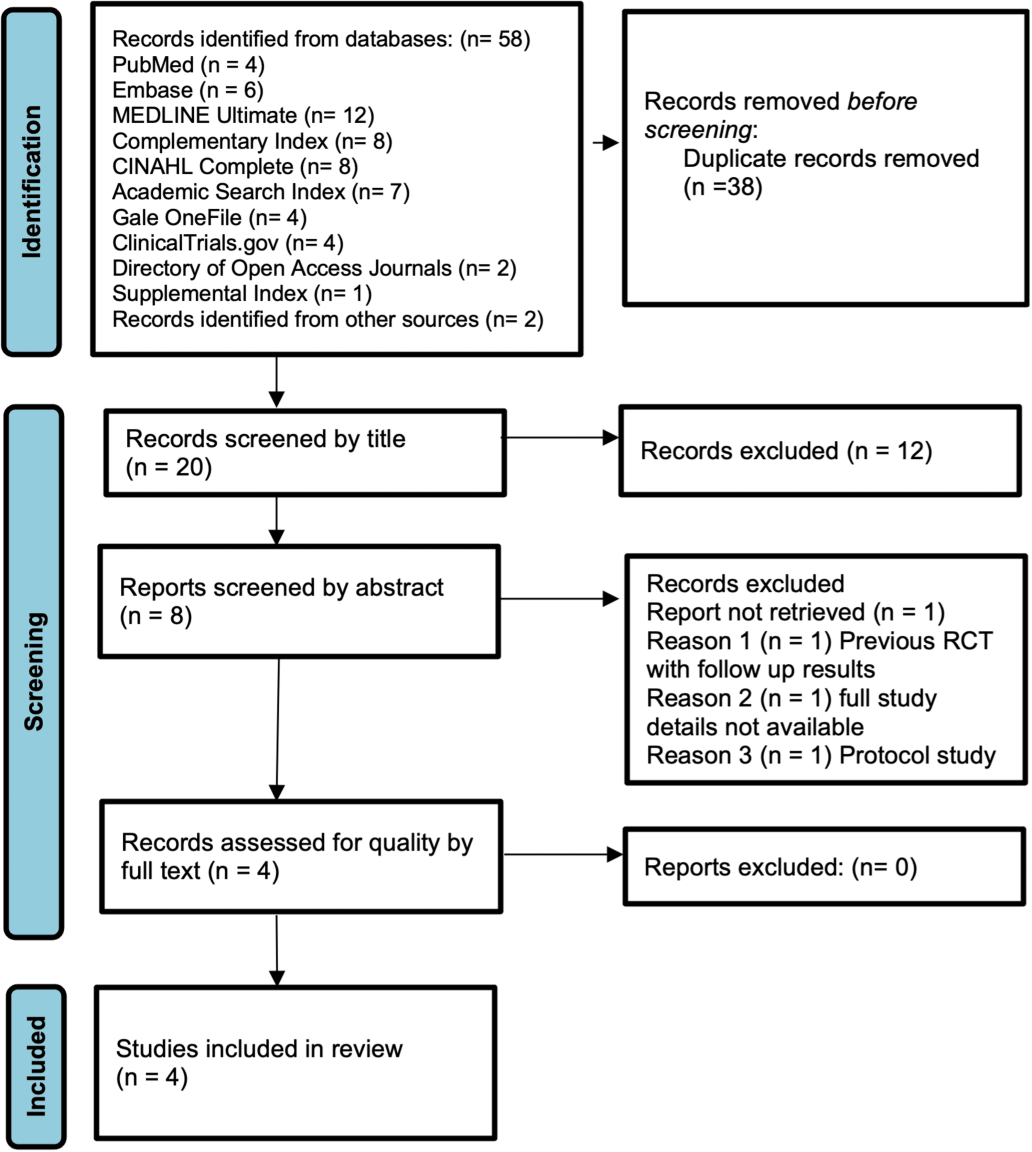
PRISMA flow diagram. PRISMA: Preferred Reporting Items for Systematic Reviews and Meta-Analyses

The systematic review initially identified 58 studies, including 56 RCTs retrieved through primary database searches and two additional studies identified via cross-referencing. Following the removal of 38 duplicates, 20 studies were screened. Of these, 12 were excluded for not meeting inclusion criteria, two lacked complete results, one was a protocol study, and one presented follow-up data from a previous trial. This rigorous selection process resulted in the inclusion of four RCTs for final analysis. The systematic screening and selection steps are visually summarised in the PRISMA flow diagram (Figure 1).

The included studies were sourced from a diverse range of databases, underscoring the comprehensiveness of the search strategy. The final studies were distributed as follows: PubMed (four studies), EMBASE (six studies), MEDLINE Ultimate (12 studies), Complementary Index (eight studies), CINAHL Complete (eight studies), Academic Search Index (seven studies), Gale OneFile: Health and Medicine (four studies), ClinicalTrials.gov (four studies), Directory of Open Access Journals (two studies), and Supplemental Index (one study). This wide-ranging coverage ensured a thorough exploration of the available literature.

The four included studies [35-38] varied in design, interventions, and follow-up durations, enrolling a total of 172 participants. Sample sizes ranged from 20 to 80 patients, with follow-up periods from two to 48 months (Table 1). These studies recruited adults with chronic lateral hip pain lasting at least three months and clinical signs of GTPS. Exclusion criteria included prior hip surgeries, recent corticosteroid injections, and systemic conditions such as diabetes or rheumatoid arthritis. Most participants were middle-aged women, reflecting the demographic most affected by GTPS. Key details of the studies, including demographic data and eligibility criteria, are summarised in Table 2.

**Table 1:** Summary of study characteristics. PRP: Platelet-Rich Plasma; RCT: Randomised Controlled Trials

Study	Study design	Level of evidence	Country	Interventions	Number of patients	Follow up in months	Results
Begkas et al. (2020) [35]	RCT	1	Greece	PRP vs corticosteroid injection	24	24	PRP better than corticosteroid
Fitzpatrick et al. (2019) [36]	RCT	1	Australia	PRP vs corticosteroid injection	80	12	PRP better than corticosteroid
Thompson et al. (2019) [37]	RCT	1	New Zealand	PRP vs placebo	48	48	PRP no better than placebo
Ribeiro et al. (2016) [38]	RCT	2	Brazil	PRP vs corticosteroid injection	20	2	PRP no better than corticosteroid

**Table 2:** Details on the number of participants, gender, age, inclusion and exclusion criteria, and intervention.

Study	Participants (n)	Gender Distribution	Average Age	Inclusion Criteria	Exclusion Criteria	Intervention Groups
Begkas et al. (2020) [35]	24	18 women, 6 men	48.7 years	Tenderness and pain over lateral hip continuously for at least 12 weeks	Recent hip injury, Inflammatory disorders, Specific conditions	Platelet-Rich Plasma (PRP) vs. Methylprednisolone
Fitzpatrick et al. (2019) [36]	80	72 women, 8 men	60 years	Lateral hip pain, Clinical signs of tendinopathy	Full-thickness tears, Previous hip surgery, Recent cortisone injections	Corticosteroid Injection (CSI) vs. Leucocyte rich (LR)-PRP
Thompson et al. (2019) [37]	48	Not specified	Not specified	Chronic lateral hip pain, Local tenderness over superior aspect of greater trochanter	Previous surgery in the area, Recent ipsilateral, corticosteroid injection, Diabetes, Rheumatoid arthritis, cardiovascular disorder, Osteoarthrosis of hip, Infection. BMI >35, high performance athletes	PRP vs placebo
Ribeiro et al. (2016) [38]	20	Not specified	18-79 years	Lateral hip pain for more than three months, Tendinobursitis diagnosed by MRI	Various medical conditions, Previous hip infiltration, Specific contraindications	PRP vs. Hexacetonide Triamcinolone

Each study employed distinct PRP preparation methods, centrifugation protocols, and injection techniques, highlighting significant variability. Blood volumes ranged from 25 mL to 54 mL, with variability in the reporting of centrifugation parameters. While some studies specified speeds as relative centrifugal force (e.g., 200 g), others reported revolutions per minute (e.g., 3,850 rpm), leading to inconsistencies and challenges in direct comparisons. Reported platelet concentrations showed a broad range, from 9.23 × 10⁹/L in Ribeiro et al. [38] to 1232 × 10⁹/L in Thompson et al. [37]. Delivery sites also differed, targeting the trochanteric bursa, gluteal tendons, or focal tender points. Ultrasound guidance was used inconsistently, with three studies employing it, while Thompson et al. [37] relied on manual techniques. These discrepancies in preparation and delivery methods are summarised in Table 3.

**Table 3:** PRP preparation and characterisation across studies. ACD-A: Anticoagulant Citrate Dextrose Solution, Solution A; PRP: Platelet-Rich Plasma

	Begkas et al. (2020) [35]	Thompson et al. (2019) [36]	Fitzpatrick et al. (2019) [37]	Ribeiro et al. (2016) [38]
Details of the kit	SW-PRP system provided by NTL Biologica	RecoverTM platelet separation collecting system (Biomet Biologics, Warsaw, Indiana, USA)	RecoverTM platelet sepa- ration collecting system (Biomet Biologics, Warsaw, Indiana, US)	Table top centrifuge
PRP spin protocol	3850 rpm for 7 min + 4 min	Centrifuged using an FDA-approved Drucker centrifuge (Biomet Biologics, Warsaw, Indiana, USA)	Centrifugal force, 1100 g; time, 15 min	15 min at 200 g in a table top centrifuge
Volume of blood taken/additives	40 mL blood + 6 mL ACD-A	54 mL blood + ACD-A 6 mL	52 mL, blood; ACD-A, 8 mL	25 mL blood + 10% citrate phosphate dextrose adenine
PRP platelet concentration	Not assessed	1232.3 x 10^9^/L (SD 637.8 x 10^9^/L)	964 x 10^9^/L (SD 551 x 10^9^/L)	9.23x10^9^/L. Mean
PRP leucocyte concentration	Not assessed	29.5 x 10^9^/L (SD, 9.0 x 10^9^/L)	35.8 x 10^9^/L (SD, 10.8 x 10^9^/L)	Not assessed
Classification of platelet	Not assessed	Not assessed	Not assessed	Not assessed
Site of delivery	Most painful place around trochanteric bursa	Focal tender point at bone depth	In the gluteal tendon	In the trochanteric bursa
Volume delivered	4 mL PRP	5 mL PRP	6-7 mL PRP	4 mL PRP
Concomitant use	None	0.3 mL of 8.4% sodium bicarbonate for buffering, 1 mL 1% xylocaine	None	0.1 mL of 10% calcium gluconate
Ultrasound used	Yes	No	Yes	Yes

Outcome measures primarily assessed pain relief and functional improvement, using tools such as the visual analogue scale (VAS) and Harris hip score (HHS). PRP demonstrated superior outcomes to corticosteroid injections in two studies (Begkas et al. [35] and Fitzpatrick et al. [36]) over 12-24 months. However, Thompson et al. [37] found no significant difference between PRP and placebo over 48 months, and Ribeiro et al. [38] reported comparable results for PRP and corticosteroids at two months. The variability in PRP preparation and administration methods complicates direct comparisons between studies but highlights PRP's potential benefits in specific contexts.

The quality of the included studies was evaluated using the Cochrane RoB 2 tool. While all studies implemented randomisation, two (Begkas et al. [35] and Ribeiro et al. [38]) showed concerns regarding allocation concealment and blinding. Fitzpatrick et al. [36] and Thompson et al. [37] exhibited a low risk of bias across all domains, while Begkas et al. [35] and Ribeiro et al. [38] were classified as having a high overall risk of bias. The risk of bias assessment results are summarised in Table 4.

**Table 4:** Risk of bias assessment across studies.

	Domain 1: Risk of bias arising from the randomization process	Domain 2: Risk of bias due to deviations from the intended	Domain 3: Missing outcome data	Domain 4: Risk of bias in measurement of the outcome	Domain 5: Risk of bias in selection of the reported result	Overall risk of bias
Ribeiro et al. [38]	Low	Low	Low	Some concerns	Some concerns	High
Fitzpatrick et al. [36]	Low	Low	Low	Low	Low	Low
Thompson et al. [37]	Low	Low	Low	Low	Low	Low
Begkas et al. [35]	Some Concerns	Some Concerns	Some Concerns	Low	Low	High

When contextualised within the broader literature, these findings align with similar issues observed in other fields of medicine using PRP. Studies in tendinopathy, osteoarthritis, and sports medicine have reported comparable variability in preparation and application protocols. Centrifugation speeds, inclusion of the buffy coat, and other procedural factors significantly influence platelet yields and growth factor concentrations, which in turn affect clinical outcomes [39-42]. Despite ongoing efforts to standardise PRP preparation across specialties, many clinical studies still fail to provide adequate details on preparation methods, leading to inconsistent results and reduced comparability [43-45]. These challenges reinforce the urgent need for detailed reporting and consensus guidelines to enable reliable assessments of PRP efficacy for various conditions, including GTPS.

For clinical practice, the variability in PRP preparation and injection protocols necessitates a tailored, patient-specific approach. Clinicians must exercise caution when interpreting the literature, given the inconsistencies in methodology and reporting. Setting realistic expectations with patients is also essential, as the heterogeneity in PRP protocols may lead to variable treatment outcomes. Until standardisation is achieved, clinical judgment and individualised care remain pivotal in optimising the use of PRP injections for GTPS.

This review has several limitations that should be considered when interpreting the findings. First, only four RCTs met the inclusion criteria, limiting the generalisability and robustness of conclusions. The small sample size and significant methodological heterogeneity across studies further complicated the synthesis of findings. Additionally, the review included only English-language studies, potentially introducing language bias and excluding relevant data from other languages. Publication bias may also have influenced the results, as studies with positive outcomes are more likely to be published. Another notable limitation is the relatively short follow-up periods in the included studies, which restrict the evaluation of PRP's long-term efficacy for GTPS.

## Conclusions

This systematic review highlights significant variability in PRP injection protocols for the management of GTPS, with notable differences in centrifugation parameters, anticoagulants, activation methods, and platelet concentrations across the included studies. Such variability affects the reproducibility of findings and complicates the establishment of evidence-based guidelines for clinical practice. While the reviewed studies generally reported positive outcomes in terms of pain relief and functional improvement, the lack of standardisation limits definitive conclusions regarding the optimal PRP preparation and administration protocols. To advance the use of PRP in GTPS management, researchers should prioritise the development of standardised preparation and reporting protocols. This includes providing detailed descriptions of centrifugation processes, platelet and leucocyte concentrations, and activation methods to enhance transparency and comparability across studies. Clinicians are encouraged to consider patient-specific factors when utilising PRP injections and set realistic expectations about treatment outcomes, given the variability in reported protocols.

Future research should focus on conducting high-quality RCTs with extended follow-up periods and harmonised methodologies to determine the most effective PRP preparation and injection strategies. Collaborative efforts to establish consensus guidelines will be crucial in bridging the current gaps, ensuring that PRP becomes a more reliable and effective treatment option for GTPS patients who have not responded to conventional therapies.

## References

[1] Silva F, Adams T, Feinstein J, Arroyo RA (2008). Trochanteric bursitis: refuting the myth of inflammation. J Clin Rheumatol.

[2] Stephens G, O'Neill S, Mottershead C, Hawthorn C, Yeowell G, Littlewood C (2020). “It’s just like a needle going into my hip, basically all of the time”. The experiences and perceptions of patients with greater trochanteric pain syndrome in the UK National Health Service. Musculoskelet Sci Pract.

[3] Fearon AM, Cook JL, Scarvell JM, Neeman T, Cormick W, Smith PN (2014). Greater trochanteric pain syndrome negatively affects work, physical activity and quality of life: a case control study. J Arthroplasty.

[4] Lievense A, Bierma-Zeinstra S, Schouten B, Bohnen A, Verhaar J, Koes B (2005). Prognosis of trochanteric pain in primary care. Br J Gen Pract.

[5] Bird PA, Oakley SP, Shnier R, Kirkham BW (2001). Prospective evaluation of magnetic resonance imaging and physical examination findings in patients with greater trochanteric pain syndrome. Arthritis Rheum.

[6] Kong A, Van der Vliet A, Zadow S (2007). MRI and US of gluteal tendinopathy in greater trochanteric pain syndrome. Eur Radiol.

[7] Kingzett-Taylor A, Tirman PF, Feller J (1999). Tendinosis and tears of gluteus medius and minimus muscles as a cause of hip pain: MR imaging findings. AJR Am J Roentgenol.

[8] Cook JL, Purdam CR (2009). Is tendon pathology a continuum? A pathology model to explain the clinical presentation of load-induced tendinopathy. Br J Sports Med.

[9] Plinsinga ML, Coombes BK, Mellor R (2018). Psychological factors not strength deficits are associated with severity of gluteal tendinopathy: a cross-sectional study. Eur J Pain.

[10] Plinsinga ML, Coombes BK, Mellor R, Vicenzino B (2020). Individuals with persistent greater trochanteric pain syndrome exhibit impaired pain modulation, as well as poorer physical and psychological health, compared with pain-free individuals: a cross-sectional study. Pain Med.

[11] Albers IS, Zwerver J, Diercks RL, Dekker JH, Van den Akker-Scheek I (2016). Incidence and prevalence of lower extremity tendinopathy in a Dutch general practice population: a cross sectional study. BMC Musculoskelet Disord.

[12] Pianka MA, Serino J, DeFroda SF, Bodendorfer BM (2021). Greater trochanteric pain syndrome: evaluation and management of a wide spectrum of pathology. SAGE Open Med.

[13] Leonard MH (1958). Trochanteric syndrome; calcareous and noncalcareous tendonitis and bursitis about the trochanter major. J Am Med Assoc.

[14] Rompe JD, Segal NA, Cacchio A, Furia JP, Morral A, Maffulli N (2009). Home training, local corticosteroid injection, or radial shock wave therapy for greater trochanter pain syndrome. Am J Sports Med.

[15] Lustenberger DP, Ng VY, Best TM, Ellis TJ (2011). Efficacy of treatment of trochanteric bursitis: a systematic review. Clin J Sport Med.

[16] Ege Rasmussen KJ, Fanø N (1985). Trochanteric bursitis. Treatment by corticosteroid injection. Scand J Rheumatol.

[17] Shbeeb MI, O'Duffy JD, Michet CJ Jr, O'Fallon WM, Matteson EL (1996). Evaluation of glucocorticosteroid injection for the treatment of trochanteric bursitis. J Rheumatol.

[18] Craig RA, Jones DP, Oakley AP, Dunbar JD (2007). Iliotibial band Z-lengthening for refractory trochanteric bursitis (greater trochanteric pain syndrome). ANZ J Surg.

[19] Slawski DP, Howard RF (1997). Surgical management of refractory trochanteric bursitis. Am J Sports Med.

[20] Verhelst L, Guevara V, De Schepper J, Van Melkebeek J, Pattyn C, Audenaert EA (2012). Extra-articular hip endoscopy: a review of the literature. Bone Joint Res.

[21] Fitzpatrick J, Bulsara M, Zheng MH (2017). The effectiveness of platelet-rich plasma in the treatment of tendinopathy: a meta-analysis of randomized controlled clinical trials. Am J Sports Med.

[22] de Vos RJ, Weir A, van Schie HT, Bierma-Zeinstra SM, Verhaar JA, Weinans H, Tol JL (2010). Platelet-rich plasma injection for chronic Achilles tendinopathy: a randomized controlled trial. JAMA.

[23] Filardo G, Kon E, Della Villa S, Vincentelli F, Fornasari PM, Marcacci M (2010). Use of platelet-rich plasma for the treatment of refractory jumper's knee. Int Orthop.

[24] Ali M, Oderuth E, Atchia I, Malviya A (2018). The use of platelet-rich plasma in the treatment of greater trochanteric pain syndrome: a systematic literature review. J Hip Preserv Surg.

[25] Mishra A, Pavelko T (2006). Treatment of chronic elbow tendinosis with buffered platelet-rich plasma. Am J Sports Med.

[26] Seijas R, Ares O, Catala J, Alvarez-Diaz P, Cusco X, Cugat R (2013). Magnetic resonance imaging evaluation of patellar tendon graft remodelling after anterior cruciate ligament reconstruction with or without platelet-rich plasma. J Orthop Surg (Hong Kong).

[27] Martinelli N, Marinozzi A, Carnì S, Trovato U, Bianchi A, Denaro V (2013). Platelet-rich plasma injections for chronic plantar fasciitis. Int Orthop.

[28] Calori GM, Tagliabue L, Gala L, d’Imporzano M, Peretti G, Albisetti W (2008). Application of rhBMP-7 and platelet-rich plasma in the treatment of long bone non-unions: a prospective randomised clinical study on 120 patients. Injury.

[29] Lee GW, Son JH, Kim JD, Jung GH (2013). Is platelet-rich plasma able to enhance the results of arthroscopic microfracture in early osteoarthritis and cartilage lesion over 40 years of age?. Eur J Orthop Surg Traumatol.

[30] Mariconda M, Cozzolino F, Cozzolino A, D'Agostino E, Bove A, Milano C (2008). Platelet gel supplementation in long bone nonunions treated by external fixation. J Orthop Trauma.

[31] Fitzpatrick J, Bulsara MK, McCrory PR, Richardson MD, Zheng MH (2017). Analysis of platelet-rich plasma extraction: variations in platelet and blood components between 4 common commercial kits. Orthop J Sports Med.

[32] Murray IR, LaPrade RF (2016). Platelet-rich plasma: renewed scientific understanding must guide appropriate use. Bone Joint Res.

[33] Dragoo JL, Wasterlain AS, Braun HJ, Nead KT (2014). Platelet-rich plasma as a treatment for patellar tendinopathy: a double-blind, randomized controlled trial. Am J Sports Med.

[34] Sterne JA, Savović J, Page MJ (2019). RoB 2: a revised tool for assessing risk of bias in randomised trials. BMJ.

[35] Begkas D, Chatzopoulos ST, Touzopoulos P, Balanika A, Pastroudis A (2020). Ultrasound-guided platelet-rich plasma application versus corticosteroid injections for the treatment of greater trochanteric pain syndrome: a prospective controlled randomized comparative clinical study. Cureus.

[36] Fitzpatrick J, Bulsara MK, O'Donnell J, Zheng MH (2019). Leucocyte-rich platelet-rich plasma treatment of gluteus medius and minimus tendinopathy: a double-blind randomized controlled trial with 2-year follow-up. Am J Sports Med.

[37] Thompson G, Pearson JF (2019). No attributable effects of PRP on greater trochanteric pain syndrome. N Z Med J.

[38] Ribeiro AG, Ricioli Junior W, Silva AR, Polesello GC, Guimarães RP (2016). PRP in the treatment of trochanteric syndrome: a pilot study. Acta Ortop Bras.

[39] Piuzzi NS, Chughtai M, Khlopas A, Harwin SF, Miniaci A, Mont MA, Muschler GF (2017). Platelet-rich plasma for the treatment of knee osteoarthritis: a review. J Knee Surg.

[40] Fadadu PP, Mazzola AJ, Hunter CW, Davis TT (2019). Review of concentration yields in commercially available platelet-rich plasma (PRP) systems: a call for PRP standardization. Reg Anesth Pain Med.

[41] Muthu S, Krishnan A, Ramanathan KR (2022). Standardization and validation of a conventional high yield platelet-rich plasma preparation protocol. Ann Med Surg (Lond).

[42] Muthuprabakaran K, Pai VV, Ahmad S, Shukla P (2021). A cross-sectional analysis of the effects of various centrifugation speeds and inclusion of the buffy coat in platelet-rich plasma preparation. Indian J Dermatol Venereol Leprol.

[43] Xu J, Du W, Xue X, Chen M, Zhou W, Luo X (2023). Global research trends on platelet-rich plasma for tendon and ligament injuries from the past two decades: a bibliometric and visualized study. Front Surg.

[44] Söderström AC, Nybo M, Nielsen C, Vinholt PJ (2016). The effect of centrifugation speed and time on pre-analytical platelet activation. Clin Chem Lab Med.

[45] Caiado A, Ferreira-Dos-Santos G, Gonçalves S, Horta L, Soares Branco P (2020). Proposal of a new standardized freeze-thawing technical protocol for leucocyte-poor platelet-rich plasma preparation and cryopreservation. Cureus.

